# Supplementary material for: 5-Fluorouracil-Immobilized Hyaluronic Acid Hydrogel Arrays on an Electrospun Bilayer Membrane as a Drug Patch
Source: Bioengineering (Basel). 2022 Nov 30;9(12):742. doi: 10.3390/bioengineering9120742 (PMC9774285; doi:10.3390/bioengineering9120742)
Supplement: Supplementary file 1 [file bioengineering-09-00742-s001.zip › Supplementary Information .pdf]

Supplementary Information

Figure S1. The image of (a) 3D-printed Vero-based master mold, and (b) replicated PDMS mold by master mold.

Figure S2. The schematic images of wettability test of electrospun bilayer membrane. (a) The attached membrane on the tilted slide glass with both surfaces facing upward in opposite positions, (b) and vice versa.

Figure S3. The schematic images of HA hydrogel arraying on electrospun bilayer membrane. (a-c) Preparation of replicated PDMS mold from Vero-based master mold. (d-e) The formed HA hydrogel array on the electrospun bilayer by ultraviolet (UV).

Figure S4. Role of bilayer membrane for mass transport as a substrate at different wettability values. The acrylic cube was filled with DI water, and the HA hydrogel array mixed with watercolor ink (blue) was placed on (a) top of the cube toward the inner parts of DI water or in (b) the opposite direction.

Figure S5. Fourier transform infrared (FT-IR) spectra of (a) PU and PU-PF surfaces of the bilayer membrane and (b) lyophilized HA.

Figure S6. Image of imperfect HA hydrogel array on PU layer (a) at a concentration of 2.5% (w/v), (b) 5% (w/v) and (c) 10% (w/v).

Figure S7. Live/dead assay images of YD-10B cells on 6-well plate dish (without HA hydrogel-arrayed bilayer membrane). (a) Control group, (b) Concentration of 1% (v/v) of 5-FU (c) concentration of 2% (v/v) of 5-FU, (d) concentration of 5% (v/v) of 5-FU and (e) Viability of YD-10B cells on 6-well plate dish for 3 days.

List of Video

Video S1. Watercolor ink (red) was dropped on the folded and trimmed membrane to compare wettability of the bilayer membrane; the PU surface locates in the upper portion and the PU-PF surface locates in bottom.

Video S2. Watercolor ink (red) was dropped on the folded and trimmed membrane to compare wettability of the bilayer membrane; the PU-PF surface locates in the upper portion and the PU surface locates in bottom.

Video S3. The relative absorption on both sides of folded bilayer membrane.
