# Supplementary figures and images for: 5-Fluorouracil-Immobilized Hyaluronic Acid Hydrogel Arrays on an Electrospun Bilayer Membrane as a Drug Patch
Source: Bioengineering (Basel). 2022 Nov 30;9(12):742. doi: 10.3390/bioengineering9120742 (PMC9774285; doi:10.3390/bioengineering9120742)

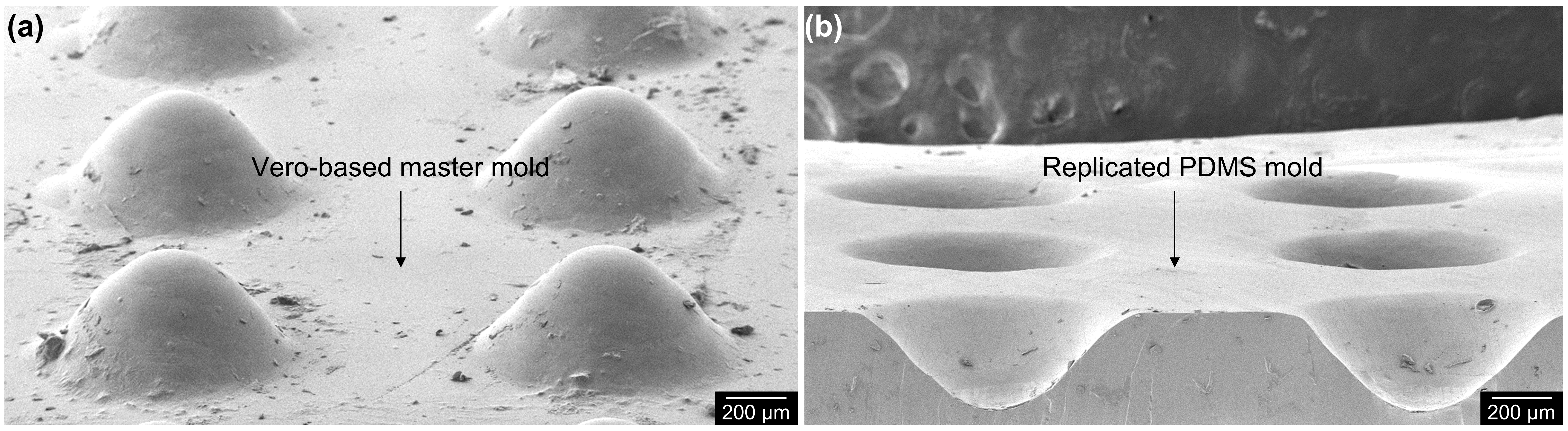

Supplement: Supplementary file 1 [file bioengineering-09-00742-s001.zip › Figure S1.TIF]

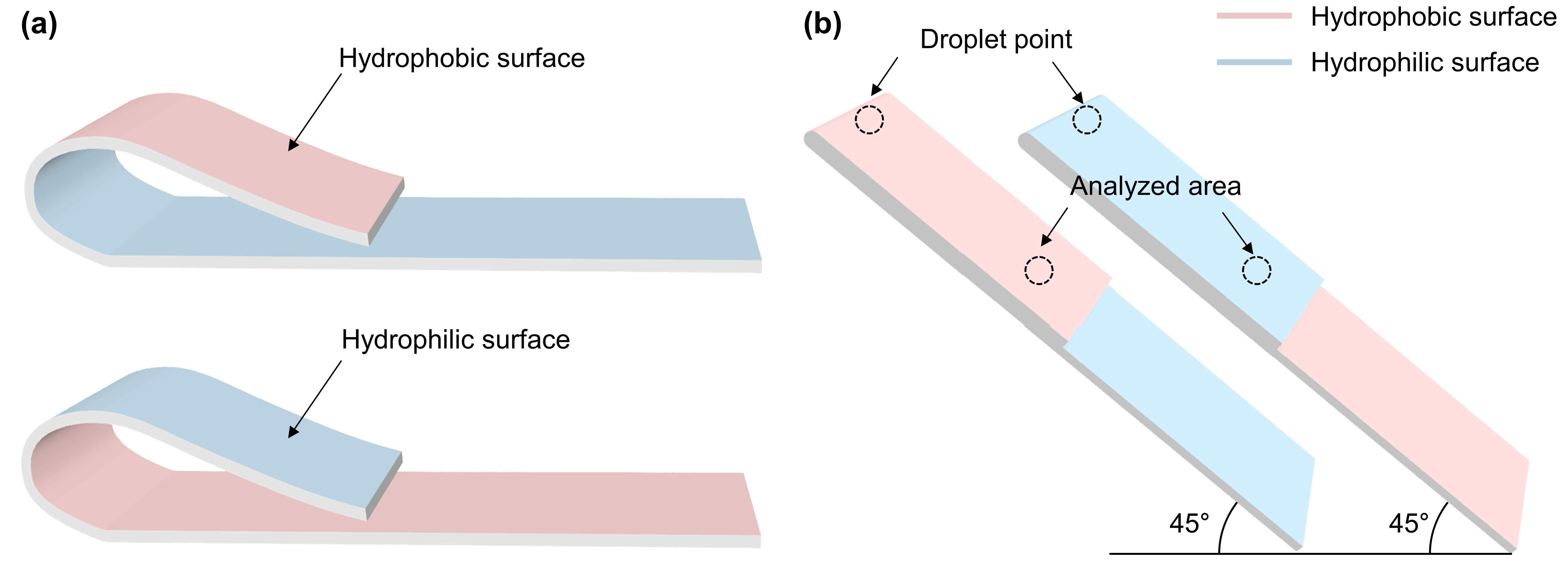

Supplement: Supplementary file 1 [file bioengineering-09-00742-s001.zip › Figure S2.TIF]

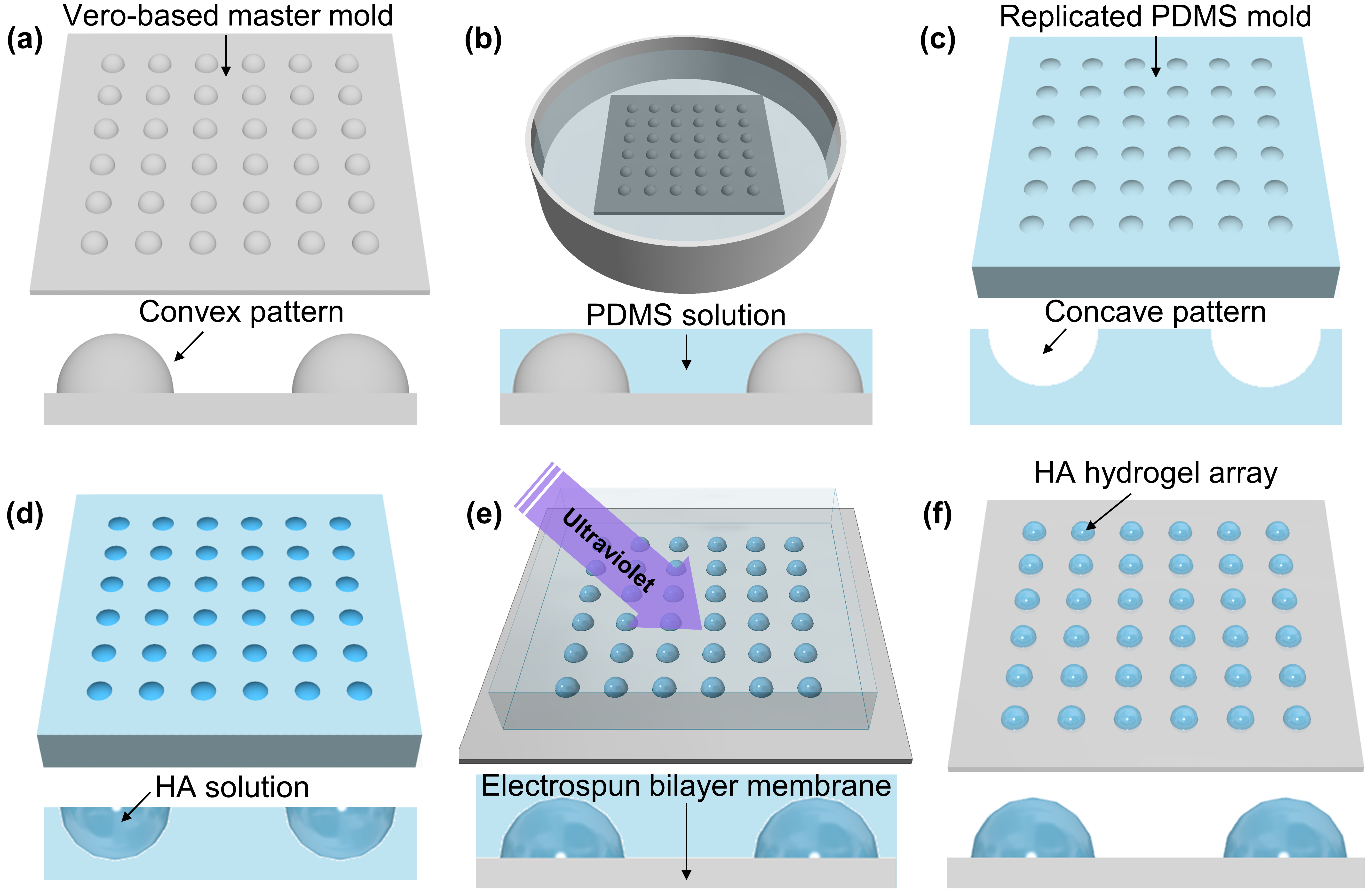

Supplement: Supplementary file 1 [file bioengineering-09-00742-s001.zip › Figure S3.TIF]

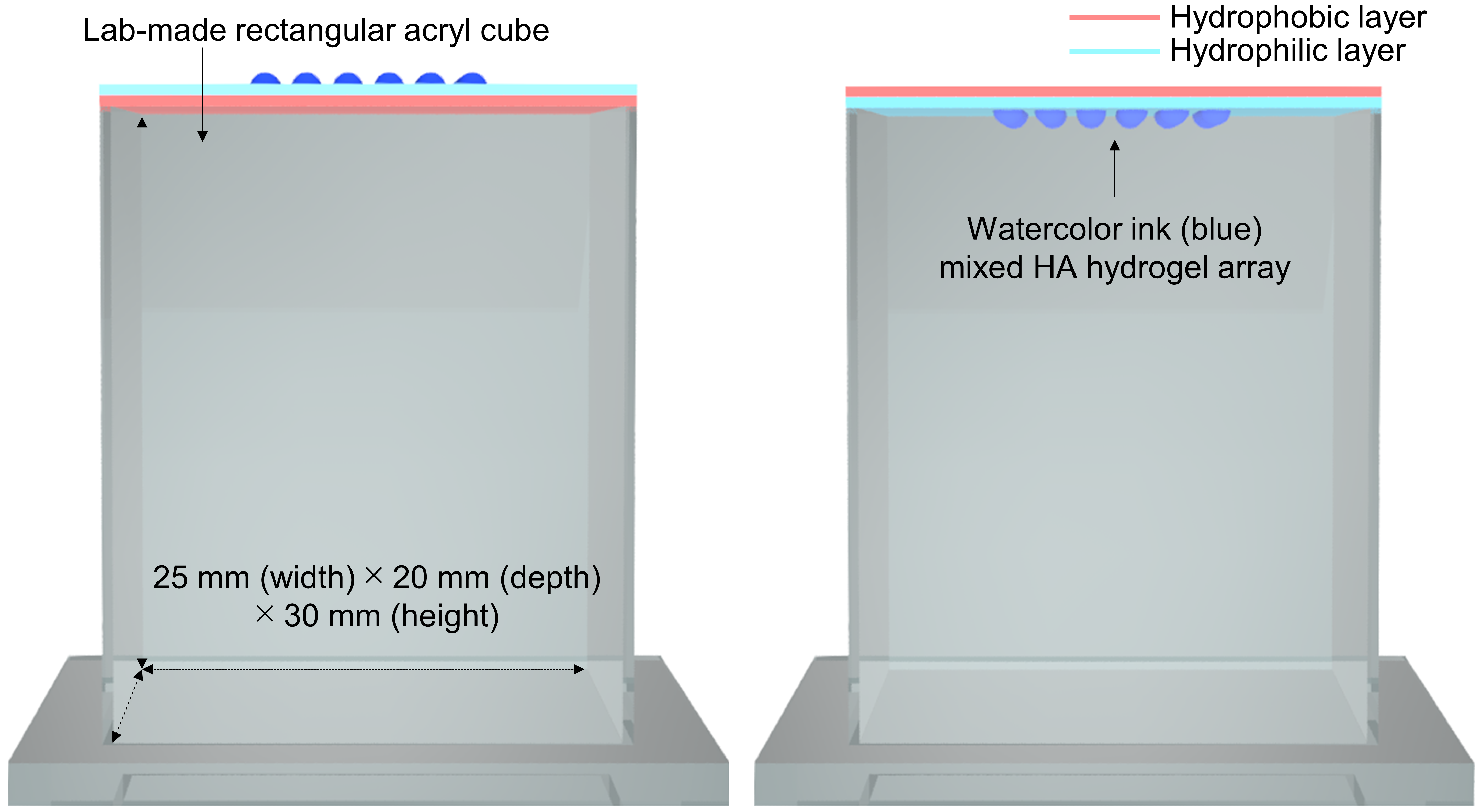

Supplement: Supplementary file 1 [file bioengineering-09-00742-s001.zip › Figure S4.TIF]

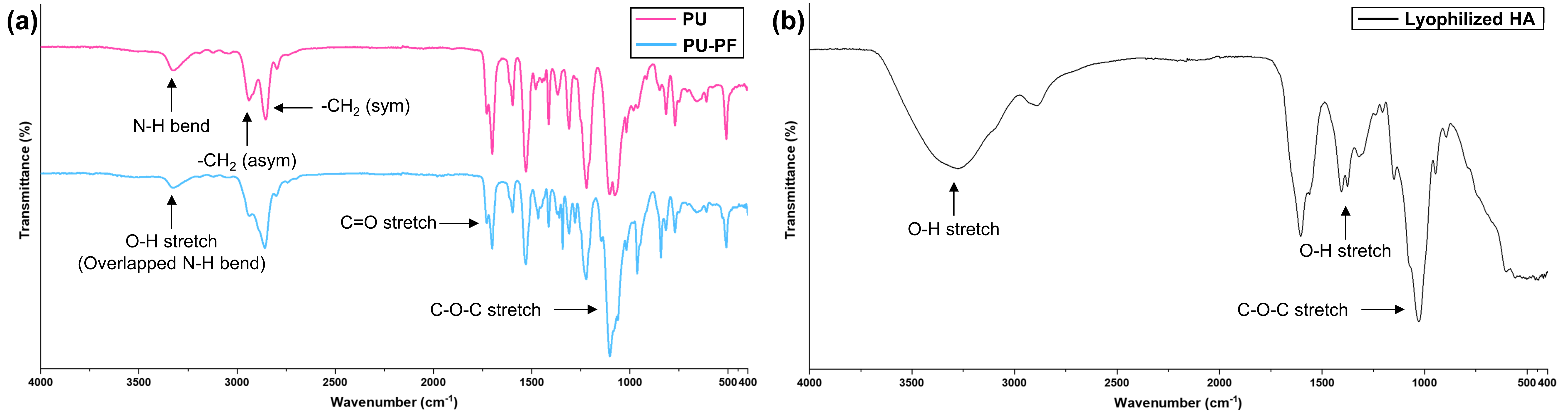

Supplement: Supplementary file 1 [file bioengineering-09-00742-s001.zip › Figure S5.TIF]

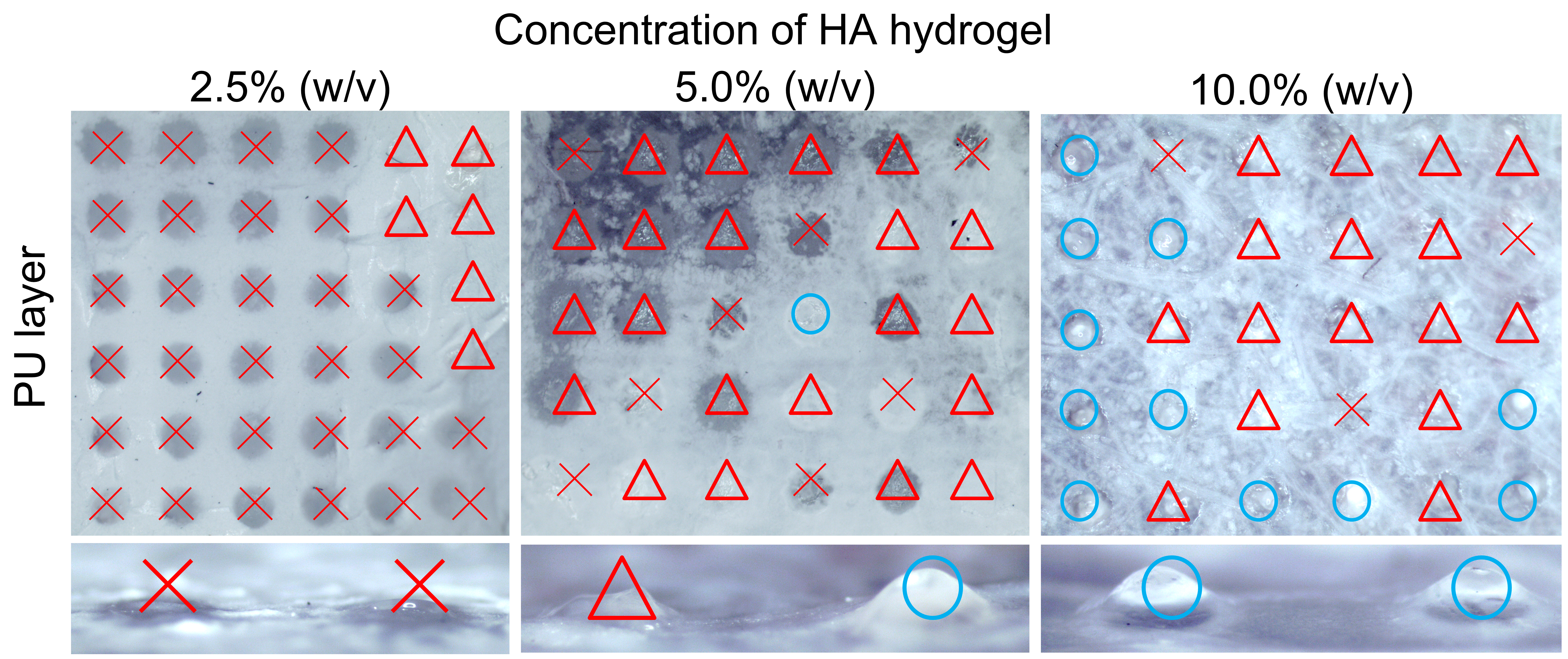

Supplement: Supplementary file 1 [file bioengineering-09-00742-s001.zip › Figure S6.TIF]

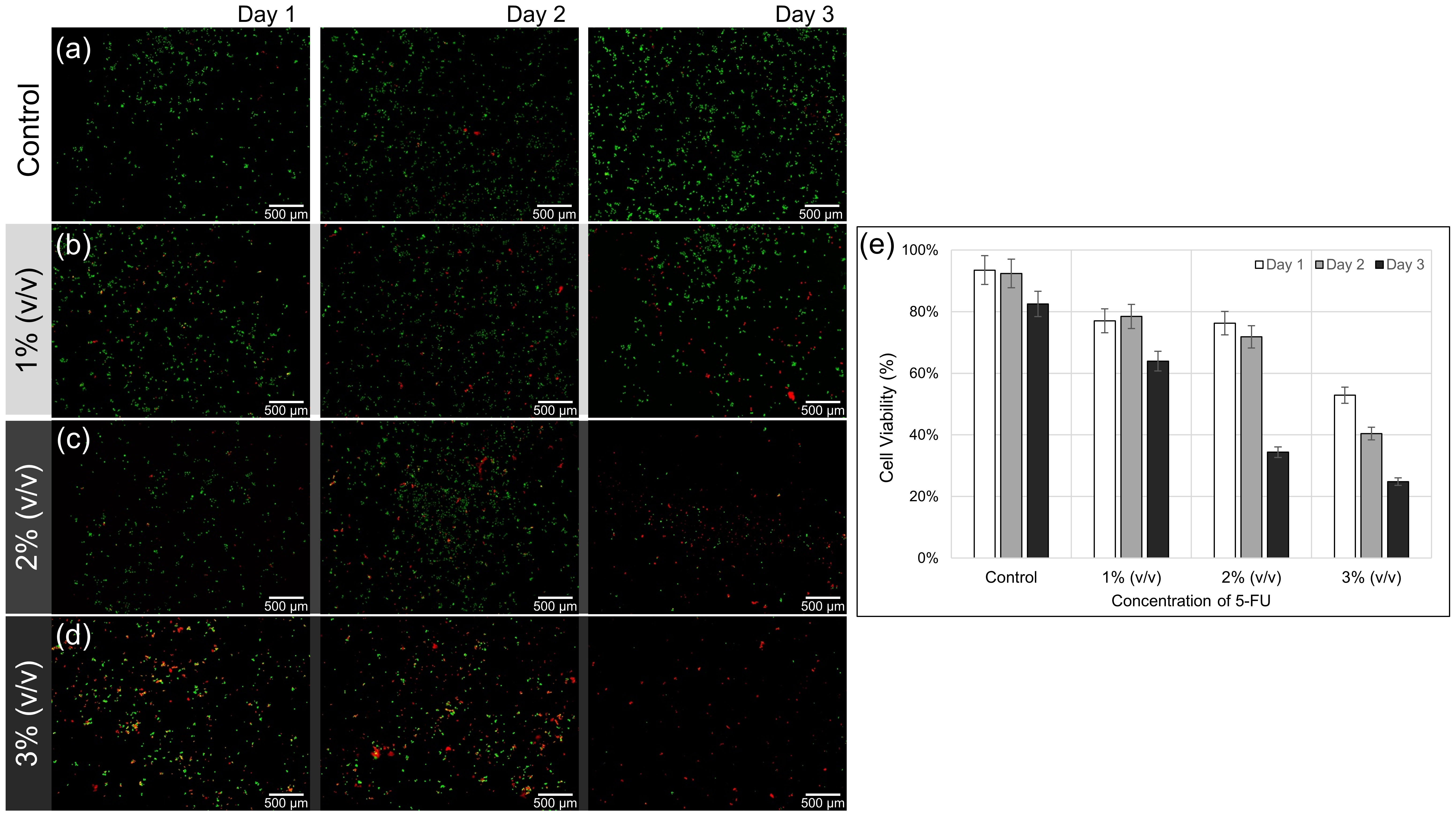

Supplement: Supplementary file 1 [file bioengineering-09-00742-s001.zip › Figure S7.TIF]
